# Single-Nucleotide Polymorphisms in Calpastatin (*CAST*) and Micro-Calpain (*CAPN1*) Genes Influencing Meat Tenderness in Crossbred Beef Cattle in Thailand

**DOI:** 10.3390/vetsci13010099

**Published:** 2026-01-19

**Authors:** Thanathip Thaloengsakdadech, Supawit Triwutanon, Preeda Lertwatcharasarakul, Nitipong Homwong, Theera Rukkwamsuk

**Affiliations:** 1 Department of Large Animal and Wildlife Clinical Sciences, Faculty of Veterinary Medicine, Kasetsart University, Kamphaeng Saen Campus, Kamphaeng Saen, Nakhon Pathom 73140, Thailand; thanathip.tha@ku.th (T.T.); supawit.tr@ku.ac.th (S.T.); 2 Department of Pathology, Faculty of Veterinary Medicine, Kasetsart University, Bangkhen Campus, Chatuchak, Bangkok 10900, Thailand; preeda.l@ku.ac.th; 3 Department of Animal Science, Faculty of Agriculture at Kamphaeng Saen, Kasetsart University, Kamphaeng Saen Campus, Kamphaeng Saen, Nakhon Pathom 73140, Thailand; nitipong.h@ku.ac.th

**Keywords:** beef tenderness, calpastatin, high-resolution melting analysis, genetic marker, single-nucleotide polymorphisms

## Abstract

This study investigated single-nucleotide polymorphisms (SNPs) within the CAPN1 316, CAPN1 4751, and CAST 2959 markers using high-resolution melting (HRM) analysis to predict meat tenderness in crossbred beef cattle. Tenderness was assessed using the Warner–Bratzler shear force (WBSF) test, with results expressed in grams (g), representing the force required to shear muscle fibers. Significant differences in phenotypic data were observed among the genotypic groups. The finding showed that polymorphisms at CAPN1 316, CAPN1 4751, and CAST 2959 exert interactive effects on meat quality traits. Notably, the TT genotype at CAPN1 4751 increased the adjusted WBSF (aWBSF) by approximately 792 g, indicating that TT was an unfavorable variant for tenderness. These results support the use of marker-assisted selection strategies in which the TT genotype is managed to minimize its frequency while other relevant markers are concurrently monitored, thereby enhancing genetic progress in meat tenderness across commercial cattle populations. This study demonstrated that CAPN1 4751 could serve as an effective marker for genetic selection in crossbred beef cattle and confirmed the efficiency of HRM analysis as a molecular tool for SNP genotyping. In conclusion, the findings provided an alternative approach for SNP detection in livestock breeding programs and represented an important step toward improving meat quality, meeting consumer expectations, and supporting the long-term sustainability of Thailand’s beef industry. The results highlighted the polygenic nature of meat tenderness and emphasized the importance of integrating multiple SNP markers to accurately assess the genetic potential for meat quality traits in cattle.

## Simple Summary

Thailand’s beef cattle industry comprises diverse production systems dominated by *Bos indicus* native breeds, Brahman crossbreds, and high-grade hybrids selected for improved growth and meat quality. Tenderness is a key consumer priority influenced by postmortem processes, proteolysis, and genetic factors, particularly single-nucleotide polymorphisms (SNPs) in the micro-calpain (*CAPN1*) and calpastatin (*CAST*) genes. In this study, qPCR-HRM (high-resolution melting) analysis was used to genotype CAPN1 316, CAPN1 4751, and CAST 2959. The CAPN1 4751 TT genotype was consistently associated with tougher meat, whereas CAST 2959 showed no phenotypic effect and CAPN1 316 had minimal influence. Population genetic analyses indicated Hardy–Weinberg equilibrium at CAPN1 316 and CAST 2959 but significant deviation at CAPN1 4751, suggesting possible selection pressures. The linkage disequilibrium (LD) analysis demonstrated predominantly independent segregation among markers, with a significant chi-squared value of LD detected between CAST 2959 and CAPN1 4751. Although crossbred genetic backgrounds and short aging times may have contributed to variation in tenderness, the findings confirmed CAPN1 4751 as a strong marker for meat quality. The polygenic nature of beef tenderness and the SNP markers detected by qPCR-HRM provide valuable tools for improving genetic selection, meat quality, and production efficiency in Thailand’s evolving beef industry.

## 1. Introduction

Thailand’s beef cattle industry is characterized by diverse population structures and production systems. In 2023, the cattle population was estimated at 9.6 million head [1]. Beef cattle raised in Thailand were originally Thai native breeds, an indigenous type characterized by small body size, short hair, and variable coat color, typically weighing between 200 and 350 kg. Based on their genetic characteristics, beef cattle in Thailand are classified into three groups. The first group, accounting for approximately 61% of the population, consists of Thai native cattle belonging to the *Bos indicus* species, including breeds such as White Lamphun, Kolan, and Kochon. These native cattle exhibit low growth rates, small body size, and relatively low carcass yields: heifers at 24 months weigh between 150 and 200 kg, steers weigh between 200 and 250 kg, and carcass yields average 32 to 33%, equivalent to roughly 50 to 60 kg per animal [2,3]. The second group consists of Brahman and Brahman-crossbred cattle, representing 35% of the national herd. These cattle are commonly used in production systems that rely on natural pastures and agricultural by-products. The third group, beef fattening cattle, includes hybrids produced by crossing Thai native or Thai Brahman cattle with *Bos taurus* breeds such as Charolais, Beefmaster, Angus, and Wagyu. These high-grade hybrids are well adapted to Thailand’s climate and exhibit faster growth rates than traditional Thai cattle, although they still grow more slowly than purebred Western breeds. Beef finishing systems in Thailand typically aim to grow cattle efficiently to market weight, relying on Angus, Beefmaster, and Wagyu crossbreds selected for marbling and growth rate.

As beef production systems evolve toward higher-value markets, improving meat quality has become a primary concern, which is influenced by attributes such as color, aroma, flavor, juiciness, and especially tenderness and intramuscular fat (marbling) [4,5,6]. However, only about 1% of cattle currently produce high-quality beef that is sold at premium prices, making meat quality improvement a key objective for producers [7]. Although low cattle prices in 2007 reduced profitability, increasing demand from China and Vietnam shifted Thailand into the role of both exporter and transit hub for regional cattle movement [4]. The cooperative production systems now play a central role, with contractual agreements among farmers, slaughterhouses, and retailers aimed at improving production capacity, processing efficiency, and supply chain management.

Among meat quality traits, tenderness is regarded as one of the most important determinants of consumer satisfaction. Meat tenderness is influenced by numerous environmental, biological, and postmortem factors, including animal age, fattening period, stress, carcass handling, cooling rate, muscle fiber type, connective tissue composition, glycogen reserves, and proteolytic activity [8,9]. Tenderness remains one of the most important attributes shaping consumer perception, yet substantial variation is still observed. The WBSF test is the standard method for evaluating tenderness, originating from early work by Warner and later refinements by Bratzler. Thresholds such as 4.1 kg have been proposed as indicators of consumer acceptability [10], although variation among studies has led to the adoption of categorical classifications (<3.0 kg, 3.0–5.7 kg, >5.7 kg) as proposed by Wheeler et al. [11]. Tenderness also varies among individual muscles, as demonstrated by Belew et al. [12]. Postmortem biochemical processes, particularly glycolysis-driven pH decline and ATP depletion, govern the development of rigor mortis and structural changes within muscle. Improper early chilling may induce cold shortening, resulting in tougher meat, whereas maintaining muscle temperature near 15 °C during rigor helps minimize excessive contraction [13]. Carcass suspension techniques can further influence sarcomere length and ultimately affect tenderness.

At the molecular level, postmortem tenderization is largely influenced by proteolysis mediated by endogenous enzymes, particularly the calpain system. This process proceeds optimally at approximately pH 6.3 and continues until enzymes lose activity [14]. The calpain system, particularly micro-calpain (*CAPN1*) and macro-calpain (*CAPN2*), regulates postmortem protein degradation, with activity modulated by the endogenous inhibitor, calpastatin (*CAST*). *CAPN1* and *CAPN2* are heterodimers composed of an 80 kDa catalytic subunit and a 28 kDa regulatory subunit, encoded on chromosomes 29 and 16, respectively. *CAST*, encoded by a complex gene with 35 exons and multiple isoforms, inhibits calpain activity until postmortem calcium accumulation disrupts this interaction, thereby activating proteolysis [15,16,17].

Genetic variability, particularly in the form of single-nucleotide polymorphisms (SNPs), plays a crucial role in the variation observed in meat tenderness. SNPs occur approximately every 700 base pairs (bp) in *Bos taurus* and every 300 bp in *Bos indicus* [18]. Several SNPs within the *CAPN1* and *CAST* genes have been identified as important markers for meat tenderness. CAPN1 316 (AF252504:g.5709C>G) in exon 14 and CAPN1 4751 (AF248054.2:g.6545C>T) in intron 17 are associated with variation in shear force, with the CC genotype at both loci consistently linked to more tender meat [19,20,21]. The CAST 2959 SNP (AF159246:g.2959G>A) located in the 3′UTR affects mRNA stability, altering *CAST* expression and thereby influencing tenderness; the A allele is associated with lower meat shear force [22,23]. These markers are already incorporated into commercial genetic tests for meat quality.

The implementation of marker-assisted selection in beef production requires efficient and accurate genotyping methods. High-resolution melting (HRM) analysis is a rapid, PCR-based method for detecting genetic variation using third-generation intercalating dyes that bind to double-stranded DNA [24]. Differences in a melting temperature (Tm) and curve profiles enable discrimination among SNP genotypes based on GC content, fragment length, and sequence composition [25]. When combined with qPCR, HRM offers enhanced precision and resolution, making it highly suitable for genotyping *CAPN1* and *CAST* markers in cattle.

The objectives of this study were to evaluate the association between gene markers (CAPN1 316 and CAPN1 4751) and the *CAST* gene marker (CAST 2959) with meat tenderness in crossbred beef cattle, and to develop quantitative PCR–high-resolution melting (qPCR-HRM) protocols for differentiating the CAPN1 316, CAPN1 4751, and CAST 2959 markers in these cattle.

## 2. Materials and Methods

### 2.1. Animals and Sample Collection

Beef samples were collected from crossbred cattle slaughtered at the Kamphaeng Saen Beef Cooperative Ltd. (Nakhon Pathom, Thailand) slaughterhouse between November 2023 and July 2024. The study population consisted of crossbred animals raised according to the cooperative’s regulations. A total of 86 crossbred cattle were included in the study. Seven-day postmortem chilled meat samples were obtained from the Longissimus Dorsi muscle (12th to 13th thoracic vertebra), transported in iceboxes to maintain the cold chain, and stored at –20 °C until analysis. Each sample was divided into two portions: one portion was processed for DNA extraction and SNP analysis, and the other portion was used to evaluate meat tenderness.

### 2.2. Molecular Analysis

For molecular analysis, genomic DNA was extracted from muscle tissue using the proteinase-K, silica membrane column method following the FavorPre^TM^ Tissue Genomic DNA Extraction Mini Kit protocol (Favorgen Biotech Corp., Taiwan, China). Primers for detecting polymorphisms at CAPN1 316, CAPN1 4751, and CAST 2959 were designed using NCBI Primer-BLAST version 2.5.0 (Table 1). qPCR-HRM reactions were prepared in a total volume of 20 μL, containing 20 ng of genomic DNA with an A260/A280 ratio of approximately 1.8, as measured using a NanoDro^TM^ 2000 Spectrophotometer (Thermo Fisher Scientific, Wilmington, DE, USA), 250 nM of each primer, 10 μL of SsoFast^TM^ EvaGreen^®^ Supermix (Bio-Rad Laboratories, Inc., Hercules, CA, USA), and RNase/DNase-free water to achieve the final volume (Table 2). PCR cycling conditions consisted of an initial denaturation at 95 °C for 4 min, followed by 40 cycles of denaturation at 95 °C for 15 s, annealing at 59.6 °C for 30 s, and extension at 72 °C for 10 s. HRM analysis was performed from 65 °C to 95 °C with 0.02 °C-per-second increments, and normalized melting curves were generated using Bio-Rad CFX Manager^TM^ 3.0 software (Bio-Rad Laboratories, Inc.) according to the manufacturer’s standard setting. Genotyping accuracy was validated by sequencing selected qPCR products using BTSeq^TM^ (Barcode-Tagged Sequencing^TM^) technology (Celemics, Inc., Seoul, Republic of Korea) by U2Bio (Thailand) Co., Ltd. (Bangkok, Thailand). The resulting sequence data were analyzed using BioEdit v7.2.5.

### 2.3. Meat Tenderness Measurement by Warner–Bratzler Shear Force Method

Meat tenderness was assessed using the Warner–Bratzler shear force (WBSF) test. Steak samples were cut to approximately 1 inch in thickness in a chilled environment, vacuum-packed, and stored at –20 °C until analysis. Before testing, frozen samples were thawed at 2–5 °C for 24 h, and 3 g of tissue was collected for DNA extraction. The remaining steak was then vacuum-repacked for cooking. Steaks were cooked in hot water at 95–100 °C until reaching an internal temperature of 71 °C, with a maximum cooking time of 30 min. After cooking, samples were cooled to 2–5 °C before coring. Subcutaneous fat and fascia were excluded. Six cylindrical cores (1.2 cm in diameter and approximately 4 cm in length) were extracted from each steak parallel to the muscle fiber orientation, one core was taken from the medial side, and five cores were taken from the lateral side of the sample. Cores that were misaligned, uneven in diameter, or contained excessive connective tissue were excluded. Shear force measurements were performed using a Brookfield AMETEK^®^ CT3 25K texture analyzer (AMETEK Brookfield, Middleboro, MA, USA). Each core was sheared once at the center, perpendicular to the muscle fibers, at a crosshead speed of 3.8 mm/sec (290 mm/min). The mean shear force of the six cores was calculated for each sample to determine meat tenderness.

### 2.4. Statistical Analysis

Allelic and genotypic frequencies of CAPN1 316, CAPN1 4751, and CAST 2959 were calculated, and the Hardy–Weinberg equilibrium (HWE) was tested using the chi-square method.

General formula of Hardy–Weinberg equation

*p*^2^ + 2*pq* + *q*^2^ = 1 (1)

The *p* represents the frequency of the dominant allele, and *q* represents the frequency of the recessive allele. *p*^2^ is the frequency of individuals dominating the homozygous dominant genotype, 2*pq* is the frequency of heterozygous individuals, and *q*^2^ is the frequency of individuals dominating the homozygous recessive genotype.

A chi-squared (*χ*^2^) was used to determine whether observed genotypic frequencies deviate significantly from the expected frequencies given HWE:

*χ*^2^ = ∑ [(*O* − *E*)^2^/*E*] (2)

where *O* is the observed frequency and *E* is the expected frequency. Degrees of freedom for the chi-squared test were calculated by the number of genotypes minus 1. A *p*-value less than 0.05 was considered to be statistically significant, suggesting deviation from HWE.

Linkage disequilibrium (LD) between markers was evaluated using *D*, *D*′, and *r*^2^ coefficients to examine non-random associations between loci.

*D*, the linkage disequilibrium estimate, is the raw difference in frequency between the observed number of AB pairs and the expected allele distributions under independence assumption. The amount of such deviation is represented by the scalar *D*, estimated as follows:

*D* = *P_AB_* − *P_A_P_B_* (3)

The LD was estimated using the raw *D*, then scaled as *D*′, a linkage disequilibrium estimate spanning the range [−1, 1].

The effect of SNP markers on meat tenderness was assessed by first fitting a linear model to adjust WBSF values for fixed effects, including marbling score, fattening period, permanent teeth, live weight, hot carcass weight, and coefficient of variation.

First fitted linear equation

*Y_ijklmno_* = *μ* + *MAR_i_* + *FAT_j_* + *TEE_k_* + *WEI_l_* + *CAR_m_* + *CV_n_* + *ε_ijklmno_* (4)

where *Y_ijklmno_* is the WBSF value, *μ* is the grand mean, *MAR* is the fixed effect of the ith marbling score, *FAT* is the fixed effect of the jth fattening period in month, *TEE* is the fixed effect of the kth effect of permanent teeth as a surrogate measure of cattle age, *WEI* is the fixed effect of the lth live weight, *CAR* is the fixed effect of the mth hot carcass weight, *CV* is the fixed effect of the nth coefficient of variation of WBSF and *ε_ijklmno_* is the random residual error for individuals oth of ith, jth, kth, lth, mth, and nth. The residuals from this first fitted linear model, referred to as the residual-adjusted model for adjusted WBSF (aWBSF) in the mixed-effect model.

After adjusting WBSF values using the first fitted linear model, a second linear model and subsequent pairwise comparison analyses were performed to evaluate the effects of the genetic markers.

The residual-adjusted model for adjusted WBSF (aWBSF) values were then used in a linear mixed effects model to evaluate the association of each genotype (CAPN1 316, CAPN1 4751, and CAST 2959) with meat tenderness, converting genotypes into dummy variables representing favorable alleles (0, 1, or 2). The linear mixed effects model is as follows:

*Y_pqrs_* = *μ* + *T*1*_p_* + *T*2*_q_* + *T*3*_r_* + *R_WBSFijklmno_* + *Z_farm_* + *ε_pqrs_* (5)

where *Y_pqrs_* is the WBSF value, *μ* is the grand mean, *T*1 is the fixed effect of the pth genotype of CAPN1 316 marker (CC = 2, CG = 1 and GG = 0), *T*2 is the fixed effect of the qth genotype of CAPN1 4751 marker (CC = 2, CT = 1 and TT = 0), *T*3 is the fixed effect of the rth genotype of CAST 2959 marker (AA = 2, AG = 1 and GG = 0), *R_WBSF_* is the fixed effect of the sth genotype of adjusted WBSF, *Z_farm_* is the random effect of farms and ε*_pqrs_* is the residual error.

All statistical analyses were performed using R version 4.5.0 [26]. The statistical significance was set at *p*-value < 0.05.

## 3. Results

### 3.1. SNPs Genotyping by HRM

The quantitative polymerase chain reaction with high-resolution melting analysis (qPCR-HRM) using the newly developed primers enabled clear discrimination between heterozygous and homozygous genotypes for the CAPN1 316, CAPN1 4751, and CAST 2959 tenderness markers. Each marker produced distinct and highly reproducible melting curve profiles across replicates, demonstrating the reliability of the method.

The amplicons generated for these markers were approximately 80–90 bp in length, indicating that the primer design effectively amplified the intended regions while minimizing non-specific amplification. The melting temperatures (Tm) were highly sensitive to genotype differences, with homozygous and heterozygous genotypes displaying clearly distinguishable melting transitions.

**Figure 1:**
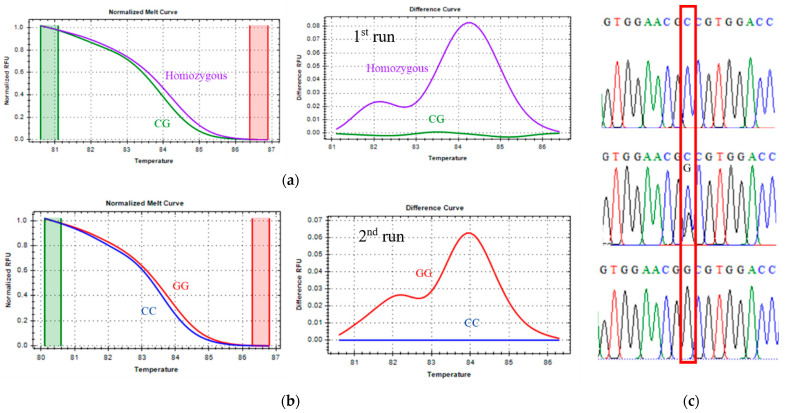
CAPN1 316 marker, melting curve (**a**,**b**), and Sanger sequencing confirmation (**c**). The first qPCR-HRM run of CAPN1 316 (**a**) was performed to discriminate between homozygous and heterozygous genotypes. The second qPCR-HRM run of CAPN1 316 (**b**) was performed only on homozygous samples to discriminate between homozygous CC and GG genotypes in the CAPN1 316 marker. The green cylindrical (**a**,**b**) represents the pre-melt phase where the DNA is fully double-stranded, the red cylindrical (**a**,**b**) represents the post-melt phase, where the DNA is fully single-stranded, and the red box (**c**) is the SNP position of interest.

**Figure 2:**
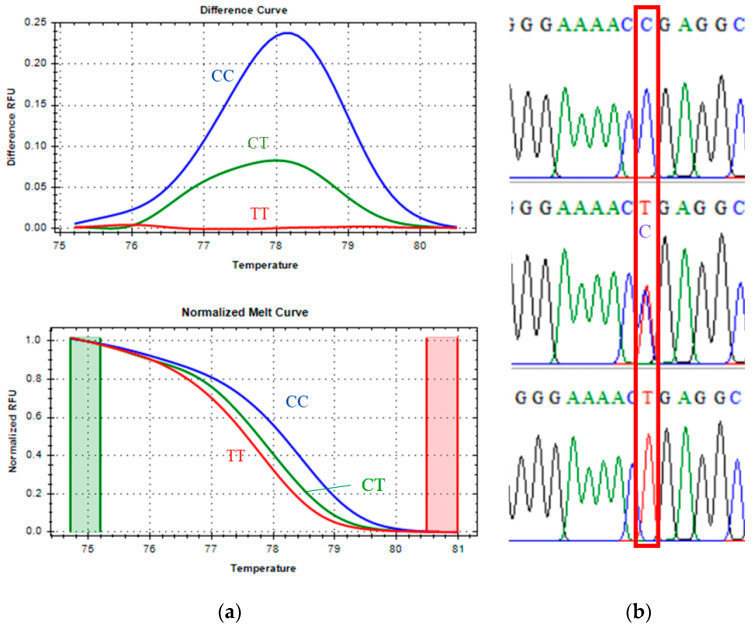
CAPN1 4751 marker, melting curve (**a**): the green cylindrical represents the pre-melt phase where the DNA is fully double-stranded, the red cylindrical represents the post-melt phase, where the DNA is fully single-stranded; and Sanger sequencing confirmation (**b**): the red box is the SNP position of interest.

**Figure 3:**
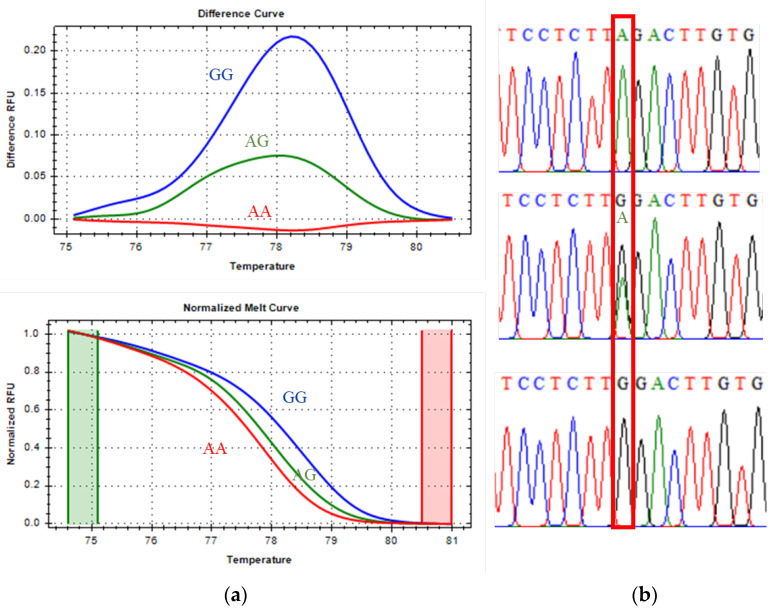
CAST 2959 marker, melting curve (**a**): the green cylindrical represents the pre-melt phase where the DNA is fully double-stranded, the red cylindrical represents the post-melt phase, where the DNA is fully single-stranded; and Sanger sequencing confirmation (**b**): the red box is the SNP position of interest.

Direct DNA sequencing of positive controls confirmed the accuracy of the genotype assignments inferred from melting curve profiles. Each genotype exhibited a unique melting transition and corresponding Tm differences, as summarized in Table 3. Selected genotypic variants were further validated by Sanger sequencing (Figure 1, Figure 2 and Figure 3), supporting the precision and robustness of the qPCR-HRM genotyping approach.

#### 3.1.1. CAPN1 316 Marker Genotyping

For the CAPN1 316 marker, classified as a class III SNP, distinct melting behaviors were observed between homozygous and heterozygous genotypes. As shown in Figure 1, the heterozygous genotype (green line) exhibited a melting profile clearly different from the homozygous genotype cluster (violet line). However, differentiating between the two homozygous genotypes—CC (favorable, blue line) and GG (unfavorable, red line)—was challenging due to their highly similar Tm values and melting curve patterns.

To address this limitation, the analysis first focused on accurately distinguishing homozygous from heterozygous samples. Subsequently, the method described by Liew et al. (2004) [27] was applied to enhance resolution between homozygous genotypes. This approach involved spiking approximately 30% wild-type homozygous DNA into the reaction mixture to induce heteroduplex formation, thereby increasing the sensitivity of the melting curve analysis. The effectiveness of this strategy in separating the CC and GG genotypes is demonstrated in Figure 1.

#### 3.1.2. CAPN1 4751 Marker Genotyping

For the CAPN1 4751 marker, classified as a class I SNP, Figure 2 shows clear discrimination among the homozygous CC (blue line), heterozygous CT (green line), and homozygous TT (red line) genotypes in both the normalized melt curves and the melting-difference curves. This distinct separation allowed accurate and straightforward genotype identification. Sanger sequencing of selected samples confirmed the presence of the expected nucleotide substitutions for each genotype, validating the reliability of the qPCR-HRM analysis for this marker.

Melting peak analysis demonstrated that the CC genotype had the highest Tm at 85.00 °C, consistent with the greater stability of C–G base pairs, which contain three hydrogen bonds. The heterozygous CT genotype had a Tm of 84.80 °C, while the TT genotype melted at 84.40 °C, reflecting the lower thermal stability of T–A base pairs with only two hydrogen bonds. The stepwise decrease in Tm was therefore consistent with the reduced number of hydrogen bonds in each genotype. Minor mutations adjacent to the SNP site were detected in some samples, as indicated by slight variations in melting profiles.

#### 3.1.3. CAST 2959 Marker Genotyping

The CAST 2959 marker, classified as a class I SNP, displayed distinct melting profiles for all three genotypes. As shown in Figure 3, the unfavorable homozygous GG genotype (blue line), the heterozygous AG genotype (green line), and the favorable homozygous AA genotype (red line) were clearly differentiated in both the melting-difference curves and the normalized melt curves. Sanger sequencing confirmed the expected nucleotide substitutions in each genotype, further validating the reliability of qPCR-HRM for CAST 2959 genotyping.

Melting peak analysis revealed that the favorable AA genotype had a Tm of 77.80 °C, the heterozygous AG genotype melted at 78.00 °C, and the unfavorable GG genotype at 78.40 °C. These differences reflect inherent variations in hydrogen bonding stability, with G–C base pairs producing higher thermal stability (and therefore higher Tm) than A–T pairs.

### 3.2. Genotype and Allele Frequencies

**Figure 4:**
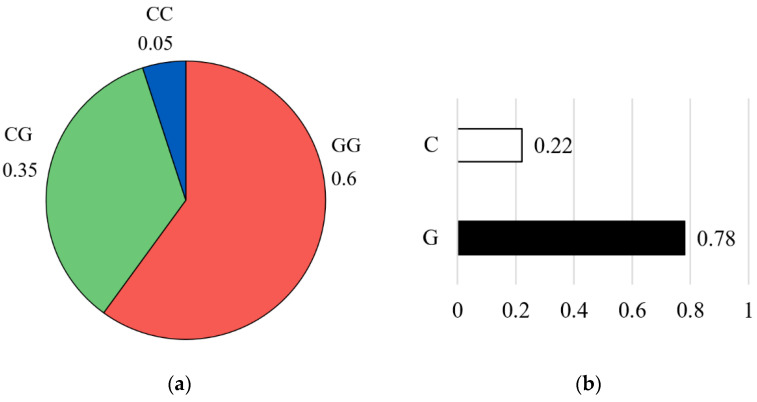
Genotype frequency (**a**) and allele frequency (**b**) for the CAPN1 316 marker. The pie chart on the left illustrates the distribution of genotypes in the studied population: the CC genotype (blue color), the CG genotype (green color), and the GG genotype (red color). The bar graph on the right shows the corresponding allele frequencies: the C allele (white color) and the G allele (black color).

For the CAPN1 316 marker, the genotype distribution showed that the homozygous GG genotype was the most prevalent, accounting for 60% of the population (n = 46), followed by the heterozygous CG genotype at 35% (n = 30), and the homozygous CC genotype at 5% (n = 4) (Figure 4a). Allele frequency analysis further indicated that the unfavorable G allele was predominant (G = 0.78), while the favorable C allele was less common (C = 0.22) (Figure 4b).

**Figure 5:**
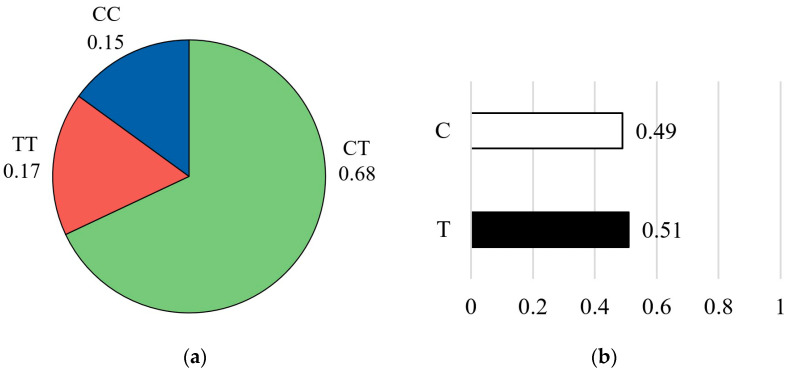
Genotype frequency (**a**) and allele frequency (**b**) for the CAPN1 4751 marker. The pie chart on the left illustrates the distribution of genotypes in the studied population: the CC genotype (blue color), the CT genotype (green color), and the TT genotype (red color). The bar graph on the right shows the corresponding allele frequencies: the C allele (white color) and the T allele (black color).

For the CAPN1 4751 marker, genotype frequencies showed that the heterozygous CT genotype was the most common, accounting for 67% of the population (n = 58). This was followed by the homozygous TT genotype at 17% (n = 15) and the homozygous CC genotype at 15% (n = 13) (Figure 5a). Allele frequency analysis revealed an almost equal distribution of the favorable C allele and the unfavorable T allele, with the T allele being slightly more frequent (T = 0.51, C = 0.49) (Figure 5b).

**Figure 6:**
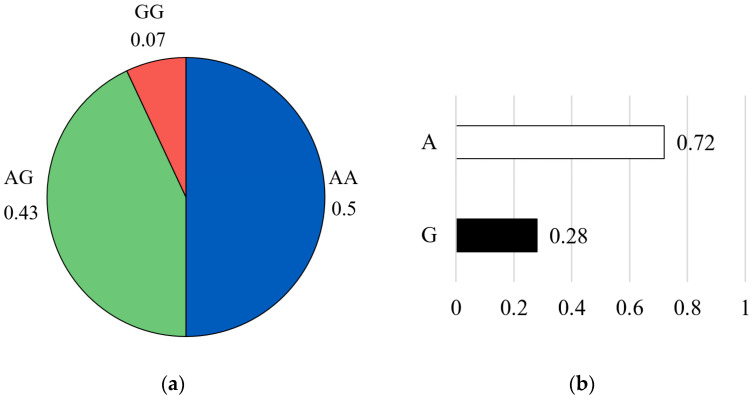
Genotype frequency (**a**) and allele frequency (**b**) for CAST 2959 marker. The pie chart on the left illustrates the distribution of genotypes in the studied population: the AA genotype (blue color), the AG genotype (green color), and the GG genotype (red color). The bar graph on the right shows the corresponding allele frequencies: the A allele (white color) and the G allele (black color).

For the CAST 2959 marker, the genotype distribution showed that the favorable homozygous AA genotype was the most common, representing approximately 50% of the population (n = 43). This was followed by the heterozygous AG genotype at 43% (n = 37), while the unfavorable homozygous GG genotype was rare, occurring in only 7% of individuals (n = 6) (Figure 6a). Allele frequency analysis further confirmed the high prevalence of the favorable A allele (A = 0.72) compared with the G allele (G = 0.28) (Figure 6b).

These results demonstrate varying distributions of favorable and unfavorable alleles across the three markers, which may have important implications for selection strategies aimed at improving meat tenderness in the studied crossbred beef population.

Table 4 summarizes the distribution of genotype combinations obtained from the three single-nucleotide polymorphism (SNP) markers—CAPN1 316, CAPN1 4751, and CAST 2959. Theoretically, a total of 27 possible genotype combinations can arise from these three loci; however, only 13 combinations were observed in the sampled population. The observed genotype combinations, along with their corresponding counts and frequencies, are presented in descending order of occurrence.

Among all detected combinations, GG/CT/AA was the most frequent, occurring in 17 samples (23%), followed by GG/CT/AG with 13 samples (18%), and CG/CT/AA with 9 samples (12%), all of which represented a substantial proportion of the population. In contrast, some genotype combinations—such as CG/TT/GG and GG/TT/GG—were rare, each occurring in only one sample (<1%).

### 3.3. Hardy–Weinberg Equilibrium

The HWE test was done for each CAPN1 316, CAPN1 4751, and CAST 2959 genotype by comparing the observed genotype counts and the expected genotype counts.

For the CAPN1 316 marker, the CC genotype had an expected count below five, making Fisher’s exact test more appropriate than Pearson’s chi-square test for assessing Hardy–Weinberg equilibrium at this locus. The test yielded a *p*-value of 1.0, indicating that the population is in equilibrium, with the observed genotype frequencies closely matching the expected values (Table 5). Overall, the G allele is the dominant allele at CAPN1 316, and the population at this marker is in HWE, with no evidence of selection, inbreeding, or other evolutionary forces acting on this locus.

The HWE test for the CAPN1 4751 locus revealed a significant deviation from equilibrium assumptions (χ^2^ = 10.51, *p* = 0.0016). The observed genotype frequencies differed markedly from those expected under HWE (Table 6). Specifically, the number of observed heterozygotes (CT) was substantially higher than expected (58.00 observed vs. 42.98 expected), whereas both homozygous genotypes were less frequent than expected (CC: 13 observed vs. 20.51 expected; TT: 15 observed vs. 22.51 expected). This significant deviation from HWE (*p* < 0.05) indicated that one or more assumptions of the Hardy–Weinberg model may be violated in this population, potentially due to non-random mating, selection pressure, genetic drift, or other evolutionary forces acting on this locus.

For CAST 2959, the observed and expected genotype counts were nearly identical (Table 7), and the *p*-value of 0.54 was much greater than 0.05. This indicated that the studied population is in Hardy–Weinberg equilibrium (HWE) at this locus. Overall, the A allele was the dominant allele at CAST 2959, and the population showed no evidence of selection, inbreeding, or other evolutionary forces acting on this marker.

### 3.4. Linkage Disequilibrium

Pairwise linkage disequilibrium (LD) analysis among the three SNP loci (CAPN1 316, CAPN1 4751, and CAST 2959) revealed varying degrees of non-random association. The LD between CAPN1 316 and CAPN1 4751 was intermediate in terms of D′ (0.2672), but showed very low allelic association (r^2^ = 0.0212), with the chi-square test indicating marginal significance (χ^2^ = 3.649, *p* = 0.056). This suggests only a weak tendency toward linkage and minimal shared allele variance between these loci. The LD between CAST 2959 and CAPN1 316 was even lower, with D′ = 0.1076 and r^2^ = 0.0083, and the association was not statistically significant (χ^2^ = 1.419, *p* = 0.234), demonstrating largely independent segregation in the population. In contrast, CAST 2959 and CAPN1 4751 exhibited the strongest evidence of LD among all marker pairs, with an intermediate D′ value (0.4731), low but higher r^2^ (0.0851), and a highly significant chi-square result (χ^2^ = 14.642, *p* = 0.00013). These results indicate a statistically non-random association in which approximately 8.5% of the variation at one locus can be explained by the other. The average pairwise LD across all three SNPs was intermediate, with a mean D′ of approximately 0.283, largely driven by the LD between CAPN1 4751 and CAST 2959, while the other pairs contributed much lower LD. The near-independence between CAPN1 316 and CAST 2959 suggests that these markers provide complementary and non-overlapping genetic information for mapping or association analyses. In contrast, the moderate LD between CAST 2959 and CAPN1 4751 may reflect physical proximity or shared selective pressures, potentially making either marker useful as a tag SNP when genotyping resources are limited. Overall, the generally intermediate LD observed among the markers supports the inclusion of all three SNPs to more comprehensively capture genetic variation related to meat quality traits.

### 3.5. Warner–Bratzler Shear Force Measurement

The Warner–Bratzler shear force (WBSF) test was used to evaluate meat tenderness in the study population (n = 86), with six replicate shear-force measurements obtained for each sample and the mean value recorded in grams. As shown in Table 8, the mean WBSF was 3868.63 g with a standard deviation of 1252.01 g, while the minimum and maximum values were 1604.70 g and 7220.00 g, respectively; the median was 3621.84 g, and the mode was 2480.00 g. The distribution of shear-force values was approximately normal, supported by the close correspondence between the mean and median and the generally symmetrical spread of the data. The interquartile range (IQR) extended from 2774.17 g (Q1) to 4913.00 g (Q3), reflecting the central tendency and variability of meat tenderness within the population. Overall, these results indicate substantial variation in WBSF values and support the assumption of normality, thereby justifying the use of parametric statistical methods for subsequent genotype–phenotype association analyses.

**Figure 7:**
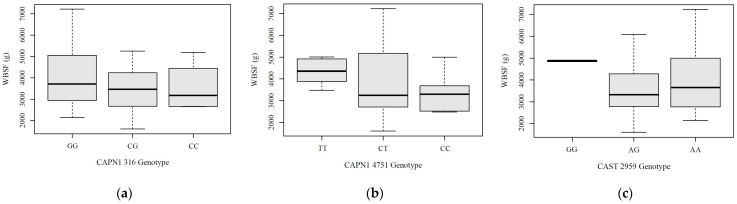
Warner–Bratzler shear force (g) distribution for each marker: (**a**) CAPN1 316 marker; (**b**) CAPN1 4751 marker; (**c**) CAST 2959 marker. The boxes represent the first quartile to the third quartile of the data, and the black bold solid lines represent the medians.

As shown in Figure 7, the association between genotypes and meat tenderness measured by WBSF revealed distinct patterns across the three markers. For CAPN1 316, the CC genotype was rare (n = 4) and exhibited a mean WBSF of 3552 g, while the CG genotype (n = 30) showed a mean WBSF of 3675 g, and the GG genotype (n = 49) had the highest mean WBSF at 4001 g, indicating a trend toward tougher beef associated with the G allele. For CAPN1 4751, the CC genotype (n = 12) had the lowest mean WBSF value at 3294 g, followed by the CT genotype (n = 57) with a mean of 3901 g, whereas the TT genotype (n = 14) exhibited the highest mean WBSF at 4191 g, suggesting a possible association between the T allele and tougher beef. For CAST 2959, the AA genotype was the most common and had a mean WBSF of approximately 3883 g with a wide range (2053.50 to 7220.00 g). The AG genotype showed a slightly lower mean WBSF of 3693 g with a similar distribution, while the GG genotype, although rare (n = 5), exhibited the highest mean WBSF at 4865 g—suggesting tougher beef, but with limited interpretive confidence due to the small sample size.

### 3.6. Significant SNP Marker Associations with Beef Tenderness

#### 3.6.1. Relationship Between Each Marker and Adjusted Warner–Bratzler Shear Force

**Figure 8:**
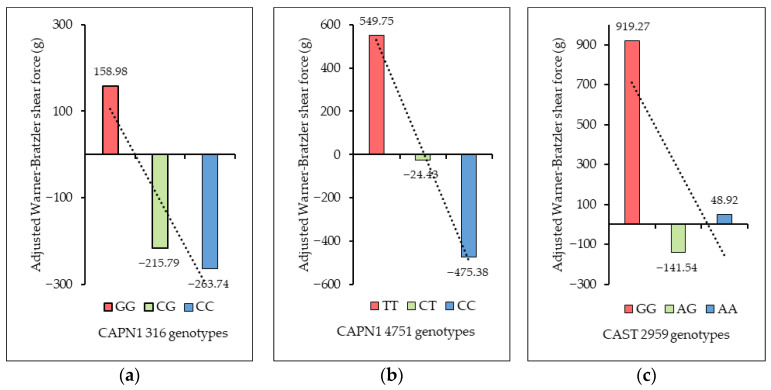
Effect of each marker on mean adjusted Warner–Bratzler shear force: (**a**) CAPN1 316 marker; (**b**) CAPN1 4751 marker; (**c**) CAST 2959 marker. The dashed lines are the trends.

As shown in Figure 8, the relationship between the genotypes of CAPN1 316, CAPN1 4751, and CAST 2959 and the adjusted Warner–Bratzler shear force (aWBSF) values revealed clear patterns in meat tenderness across all three markers. For CAPN1 316, the unfavorable GG genotype exhibited the highest mean aWBSF (158.98 g), followed by the CG genotype (–215.79 g) and the favorable CC genotype (–263.74 g), with corresponding standard deviations of 1333.36 g, 989.61 g, and 1150.50 g, respectively—showing a decreasing trend in aWBSF from GG to CC. For CAPN1 4751, the unfavorable TT genotype showed the highest mean aWBSF (549.75 g), followed by the CT genotype (−24.43 g) and the favorable CC genotype (−475.38 g), with standard deviations of 648.44 g, 1335.80 g, and 726.31 g, respectively, again demonstrating a decreasing pattern from TT to CC. For CAST 2959, the unfavorable GG genotype had the highest mean aWBSF (919.27 g), followed by AA (48.92 g), while the AG genotype exhibited the lowest mean aWBSF (−141.54 g), with standard deviations of 489.97 g, 1112.19 g, and 1302.62 g, respectively—indicating a decreasing trend from GG to AG, with a slight increase at AA. Overall, these patterns suggest that favorable alleles across the three loci were consistently associated with lower adjusted WBSF values and therefore improved meat tenderness.

#### 3.6.2. Significant Marker Combination Associations with Beef Tenderness

After adjusting WBSF values using the first fitted linear model and conducting a second linear model with pairwise comparison analysis, the results indicate that allelic substitution at the CAPN1 4751 locus exerts the strongest and most consistent influence on meat tenderness among the markers evaluated. As shown in Table 9, a significant association was detected between the CG/TT/AA and CG/CC/AA genotype combinations (CAPN1 316/CAPN1 4751/CAST 2959), with a substantial difference of 792.24 ± 207.98 g in WBSF values (*p* = 0.02). This demonstrates that animals carrying the CG/TT/AA combination produce significantly tougher meat than those with the CG/CC/AA combination. Two additional contrasts in which TT animals were compared with CC or CT genotypes at CAPN1 316 yielded similar effect sizes (523.34 ± 172.62 g and 565.89 ± 187.72 g, respectively), although their *p*-values (0.052–0.056) indicated marginal statistical significance.

**Figure 9:**
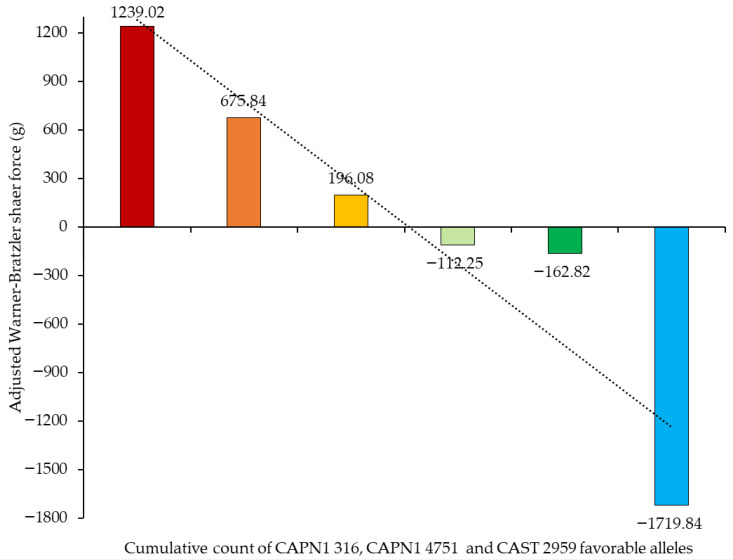
Effect of cumulative count of CAPN1 316, CAPN1 4751, and CAST 2959 favorable alleles on adjusted Warner–Bratzler shear force. (■ = 0 favorable allele, ■ = 1 favorable allele, ■ = 2 favorable alleles, ■ = 3 favorable alleles, ■ = 4 favorable alleles, ■ = 5 favorable alleles). The dashed line represents the trend.

Figure 9 demonstrates the association between the cumulative count of favorable alleles of the CAPN1 316, CAPN1 4751, and CAST 2959 markers and the corresponding aWBSF values. A clear negative trend was observed, indicating that as the number of favorable alleles increased, aWBSF values decreased. This pattern suggests that favorable alleles at these loci exert an additive effect on improving meat tenderness. Beef samples with no favorable alleles exhibited the highest aWBSF values, whereas those carrying five favorable alleles showed the lowest value, suggesting a cumulative genetic effect of *CAPN1* and *CAST* on reducing shear force and enhancing beef tenderness.

## 4. Discussion

In this study, we used qPCR-HRM to genotype SNPs at CAPN1 316, CAPN1 4751, and CAST 2959 loci, which were associated with beef tenderness. Our results indicated that the TT genotype of CAPN1 4751 was a consistent indicator of tougher beef, although CAPN1 4751 located in an intronic region which could be regulate gene expression and alternative splicing, it may influence the activity of the calpain system, thereby affecting postmortem proteolysis and tenderization, as evidenced by significantly higher WBSF values, making it a reliable marker for tenderness prediction. In contrast, the CAST 2959 locus showed no significant phenotypic effect in the studied population. Although the combination of CAPN1 4751 and CAPN1 316 appeared to exert some influence on tenderness, the evidence remained inconclusive. These findings aligned with previous studies by Van Eenennaam et al. [28] and White et al. [29], which also reported strong associations between the CAPN1 4751 and beef tenderness. Interpretation of these genetic effects must also account for pedigree-related factors, as the crossbred cattle population used in this study likely varied in their proportion of *Bos taurus* and *Bos indicus* ancestry, which differed in both SNP distribution and phenotypic expression. Such genetic heterogeneity added complexity to marker evaluation and highlighted the importance of larger sample sizes or detailed pedigree information to improve the accuracy of SNP effect estimates. It should be noted that the sample size in this study was limited to 86 individuals, based on available resources. While larger sample sizes are generally preferable for detecting small-to-medium effect sizes with higher statistical power, the current sample size reflects practical constraints. As a result, the findings should be interpreted with caution, given the increased risk of Type II error and potentially reduced statistical power. Additionally, slaughtering, aging, and cooking conditions can influence meat tenderness. Although all samples were collected from a single slaughterhouse to minimize environmental variability, the relatively short 7-day aging period may have reduced proteolytic enzyme activity and limited the detectable influence of genetic differences compared with longer 14–21-day aging periods commonly used in tenderness studies [30]. The excess of heterozygous genotype observed at the CAPN1 4751 locus suggests that dominance effects may contribute to variation in meat tenderness, as the aWBSF of heterozygous genotypes appears to be closer to the favorable allele than to the unfavorable allele. Although the present study was restricted to additive genetic models, this observation highlights the need for future studies incorporating dominance effects to fully characterize the role of CAPN1 polymorphisms, particularly in crossbred populations. For the CAPN1 316 locus, the requirement for a second HRM run with DNA spiking highlights a limitation of the qPCR-HRM method in detecting certain SNP classes, particularly class III SNPs due to the formation of double-stranded DNA stabilized by an equal number of hydrogen bonds resulting in minimal differences in melting temperature, combined with the resolution limits of our qPCR-HRM instrument may further restrict the discriminatory ability of this technique. Although this additional step enables accurate genotyping, it increases processing time and labor, potentially limiting throughput in routine or large-scale applications. Furthermore, the sensitivity of HRM for detecting subtle melting differences can vary among instrument models; therefore, the use of high-performance qPCR-HRM systems in combination with optimized fluorescent dyes is recommended to improve resolution and reliability for these challenging SNPs. These considerations underscore the importance of validating HRM protocols for each target SNP and instrument setup prior to integration into commercial genotyping pipelines or high-throughput breeding programs.

The HWE analysis provided additional insight into population structure. The CAPN1 316 locus showed genotype frequencies consistent with HWE, and Fisher’s exact test confirmed equilibrium despite the small expected count for the CC genotype, suggesting that random mating rather than selection was shaping allele distribution at this locus. The sample size (n = 86), although modest, was sufficient to detect moderate deviations and support the interpretation of genetic stability at CAPN1 316. In contrast, CAPN1 4751 significantly deviated from HWE (χ^2^ = 10.51), showing an excess of CT heterozygotes and fewer CC and TT homozygotes than expected. This pattern may indicate heterozygote advantage, non-random mating, genetic drift, or sampling error; however, the consistent overrepresentation of heterozygotes suggested that evolutionary forces such as selection could be acting on this locus. A larger and more diverse sample was needed to determine whether selection was influencing CAPN1 4751 allele frequencies. Meanwhile, the CAST 2959 locus remained in equilibrium, with observed and expected genotype counts nearly identical, indicating that allele A is stable in the population and not strongly affected by selection or inbreeding. Although the relatively small sample size increases the likelihood of false negatives, the clear deviation observed only at CAPN1 4751 suggested that different evolutionary pressures may be influencing each of the three loci.

The analysis of LD revealed that the CAST 2959 × CAPN1 4751 pair exhibited a low level of association (*r*^2^ = 0.0851) but a moderate degree of linkage disequilibrium (D′ = 0.4731), accompanied by a highly significant chi-squared value (χ^2^ = 14.642). Despite this, the negative correlation (*r* = −0.2918) indicated an inverse allelic relationship, in which the minor allele of CAPN1 4751 was frequently associated with the major allele of CAST 2959. Such patterns may reflect historical recombination events or selective breeding pressures that have shaped current haplotype structures. In contrast, LD among the remaining marker pairs was low, and the average D′across all pairs (approximately 0.283) indicated overall weak to moderate linkage, supporting the absence of strong linkage blocks and suggesting that each SNP contributes independent genetic information. This independence is advantageous for association mapping, particularly between CAPN1 316 and CAST 2959, while the significant chi-square value for CAPN1 4751 and CAST 2959 suggested that a tag SNP may be useful when resources are limited. Collectively, the LD patterns support the use of all three SNPs to capture broader genetic variation associated with meat quality traits.

Moreover, the study demonstrated that qPCR-HRM was an efficient, sensitive, and cost-effective method for SNP genotyping, provided that primer design was sufficiently specific to prevent the formation of multiple melting domains and that sequencing-verified positive controls were included to ensure a positive result. The qPCR-HRM is applicable to tissue, blood, or semen samples, making it suitable for integration into commercial breeding programs. Looking forward, although CAPN1 4751 emerged as a strong candidate marker for beef tenderness, incorporating additional SNPs associated with other meat quality traits and exploring multiplex qPCR-HRM could further enhance the precision of genetic selection and the efficiency of high-throughput screening. Future research should also assess economic feasibility and conduct longitudinal studies to evaluate the long-term effects of selection based on CAPN1 316, CAPN1 4751, and CAST 2959 on meat quality improvement and overall cattle production profitability.

## 5. Conclusions

In conclusion, the qPCR-HRM represents a promising and efficient approach for improving beef tenderness. The qPCR-HRM approach developed in this study represents an efficient and practical tool for genetic improvement programs in beef cattle. When integrated with other genomic technologies, this method offers a valuable pathway to enhance meat quality, breeding efficiency, and long-term sustainability of livestock production systems. The findings reinforce the polygenic nature of meat tenderness and highlight the importance of applying multiple genetic markers simultaneously rather than relying on single loci. Such strategies are particularly relevant for beef cattle populations in Thailand, where genomic-assisted selection can contribute substantially to improved productivity and competitiveness of the beef industry.

## Figures and Tables

**Table 1:** qPCR primers and product size to detect polymorphism of CAPN1 316, CAPN1 4751, and CAST 2959 markers.

SNP	Accession	Primer		Annealing Temperature (°C)	Amplicon (bp)
CAPN1 316	AF 252504	Forward	5′-CAGCTCCTCG	59.60	76
			GAGTGGAAC-3′		
		Reverse	5′-AACTCCCCAT	59.60	
			CCTCCATCTT-3′		
CAPN1 4751	AF 248054	Forward	5′-CTGGCATCCT	59.60	86
			CCCCTTGACT-3′		
		Reverse	5′-GGGTCACCTG	59.60	
			TAGACGAGGC-3′		
CAST 2959	AF 159246	Forward	5′-CGATGCCTCAC	59.60	77
			GTGTTCTTC-3′		
		Reverse	5′-ACATCAAACAC	59.60	
			AGTCCACAAGT-3′		

**Table 2:** qPCR component for reaction setup.

qPCR Component	Volume Per Reaction	Concentration Per Reaction
SsoFast^^TM^^ EvaGreen^®^ Supermix	10 µL	1x
Forward primer	0.5 µL	250 nM
Reverse primer	0.5 µL	250 nM
RNase/DNase-free water	8 µL	-
DNA template	1 µL	20 ng
Total volume	20 µL	

**Table 3:** Melting temperature of genotypes for CAPN1 316, CAPN1 4751, and CAST 2959 markers.

Markers	Genotypes	Melting Temperature (°C)
CAPN1 316	CC	84.20 (83.60 *)
	CG	84.00
	GG	84.20 (83.80 *)
CAPN1 4751	CC	85.00
	CT	84.80
	TT	84.40
CAST 2959	AA	77.80
	AG	78.00
	GG	78.40

* Second run was performed to discriminate only homozygous genotypes in CAPN1 316 marker.

**Table 4:** Genotype distribution for CAPN1 316, CAPN1 4751, and CAST 2959 combinations.

CAPN1 316/CAPN1 4751/CAST 2959 Genotypes Combination	Count	Frequency
GG/CT/AA	17	0.23
GG/CT/AG	13	0.18
CG/CT/AA	9	0.12
CG/CT/AG	8	0.11
CC/CT/AG	4	0.05
CG/CC/AA	4	0.05
GG/CC/AA	4	0.05
GG/TT/AG	4	0.05
CG/CC/AG	3	0.04
CG/TT/AA	3	0.04
GG/TT/AA	3	0.04
CG/TT/GG	1	0.01
GG/TT/GG	1	0.01

**Table 5:** Observed and expected genotype counts for the CAPN1 316 genotype.

CAPN1 316 Genotype	Observed Count	Expected Count
CC (favorable)	4	4.20
CG	30	29.60
GG (unfavorable)	52	52.20

**Table 6:** Observed and expected genotype counts for the CAPN1 4751 genotype.

CAPN1 4751 Genotype	Observed Count	Expected Count
CC (favorable)	13	20.51
CT	58	42.98
TT (unfavorable)	15	22.51

**Table 7:** Observed and expected genotype counts for the CAST 2959 genotype.

CAST 2959 Genotype	Observed Count	Expected Count
AA (favorable)	43	43.98
AG	37	35.04
GG (unfavorable)	6	6.98

**Table 8:** The summary statistics for Warner–Bratzler shear force.

Descriptive Data	WBSF (g)
Count	86
Mean WBSF	3868.63
Standard deviation	1252.01
Mode	2480.00
Min	1604.67
25%	2774.17
50%	3621.84
75%	4914.00
Max	7220.00

**Table 9:** Significant marker combination associations with beef tenderness.

Marker Combination	Pairwise Comparison	Estimate Different	SE	*p*-Value
CAPN1 316/CAPN1 4751/CAST 2959	CG/TT/AA−CG/CC/AA	792.24	207.98	0.02
CAPN1 316/CAPN1 4751	CG/TT−CG/CC	523.34	172.62	0.05
CAPN1 316/CAPN1 4751	CG/TT−CC/CT	565.89	187.73	0.06

## Data Availability

The original contributions presented in this study are included in the article. Further inquiries can be directed to the corresponding author.

## Abbreviations

The following abbreviations are used in this manuscript:

aWBSF	Adjusted Warner–Bratzler shear force
bp	Base pairs
*CAPN1*	Micro-Calpain
*CAST*	Calpastatin
DNA	Deoxyribonucleic acid
DNase	Deoxyribonuclease
g	Gram
HRM	High-resolution melting analysis
kg	Kilogram
PCR	Polymerase chain reaction
qPCR	Quantitative polymerase chain reaction
RNA	Ribonucleic acid
RNase	Ribonuclease
Tm	Melting temperature
μL	Microliter
μM	Micromolar
mg	Milligram
pH	Potential of hydrogen
SNPs	Single-nucleotide polymorphisms
WBSF	Warner–Bratzler shear force

## References

[1] Department of Livestock Development (DLD) Livestock Statistics of Thailand 2023. https://ict.dld.go.th/index.php/th/service/report/book/report-book-2566.

[2] Wangkumhang P., Wilantho A., Shaw P.J., Flori L., Moazami-Goudarzi K., Gautier M., Duangjinda M., Assawamakin A., Tongsima S. (2015). Genetic analysis of Thai cattle reveals a Southeast Asian indicine ancestry. PeerJ.

[3] Yodsoi S., Chaiwang N., Yammuen-art S., Sringarm K., Suwansirikul S., Jaturasitha S. (2013). Meat quality of Mountainous and white Lamphun cattle compared to Brahman crossbred. Khon Kaen Agric. J..

[4] Bunmee T., Chaiwang N., Kaewkot C., Jaturasitha S. (2018). Current situation and future prospects for beef production in Thailand—A review. Asian-Australas. J. Anim. Sci..

[5] Henchion M.M., McCarthy M., Resconi V.C. (2017). Beef quality attributes: A systematic review of consumer perspectives. Meat Sci..

[6] Miller R. (2020). Drivers of consumer liking for beef, pork, and lamb: A review. Foods.

[7] Shackelford S.D., Wheeler T.L., Meade M.K., Reagan J.O., Byrnes B.L., Koohmaraie M. (2001). Consumer impressions of tender select beef. J. Anim. Sci..

[8] Špehar M., Vincek D., Žgur S. (2008). Beef quality: Factors affecting tenderness and marbling. Stočarstvo.

[9] Raza S.H.A., Kaster N., Khan R., Abdelnour S.A., El-Hack M.E.A., Khafaga A.F., Taha A., Ohran H., Swelum A.A., Schreurs N.M. (2020). The role of microRNAs in muscle tissue development in beef cattle. Genes.

[10] Huffman K.L., Miller M.F., Hoover L.C., Wu C.K., Brittin H.C., Ramsey C.B. (1996). Effect of beef tenderness on consumer satisfaction with steaks consumed in the home and restaurant. J. Anim. Sci..

[11] Wheeler T., Shackelford S., Koohmaraie M. (1997). Standardizing collection and interpretation of Warner-Bratzler shear force and sensory tenderness data. Proc. Recip. Meat Conf..

[12] Belew J.B., Brooks J.C., McKenna D.R., Savell J.W. (2003). Warner–Bratzler shear evaluations of 40 bovine muscles. Meat Sci..

[13] Devine C.E., Wahlgren N.M., Tornberg E. (1999). Effect of rigor temperature on muscle shortening and tenderisation of restrained and unrestrained beef m. longissimus thoracicus et lumborum. Meat Sci..

[14] Dransfield E. (1994). Optimisation of tenderisation, ageing and tenderness. Meat Sci..

[15] Raynaud P., Gillard M., Parr T., Bardsley R., Amarger V., Levéziel H. (2005). Correlation between bovine calpastatin mRNA transcripts and protein isoforms. Arch. Biochem. Biophys..

[16] Campbell R.L., Davies P.L. (2012). Structure–function relationships in calpains. Biochem. J..

[17] Leveau C. (2008). Candidate Genes for Beef Quality—Allele Frequencies in Swedish Beef Cattle. Master’s Thesis.

[18] Seidel G.E. (2009). Brief introduction to whole-genome selection in cattle using single nucleotide polymorphisms. Reprod. Fertil. Dev..

[19] Page B.T., Casas E., Heaton M.P., Cullen N.G., Hyndman D.L., Morris C.A., Crawford A.M., Wheeler T.L., Koohmaraie M., Keele J.W. (2002). Evaluation of single-nucleotide polymorphisms in *CAPN1* for association with meat tenderness in cattle. J. Anim. Sci..

[20] Casas E., White S.N., Riley D.G., Smith T.P.L., Brenneman R.A., Olson T.A., Johnson D.D., Coleman S.W., Bennett G.L., Chase C.C. (2005). Assessment of single nucleotide polymorphisms in genes residing on chromosomes 14 and 29 for association with carcass composition traits in *Bos indicus* cattle. J. Anim. Sci..

[21] Curi R.A., Chardulo L.A.L., Mason M.C., Arrigoni M.D.B., Silveira A.C., de Oliveira H.N. (2009). Effect of single nucleotide polymorphisms of *CAPN1* and *CAST* genes on meat traits in Nellore beef cattle (*Bos indicus*) and in their crosses with *Bos taurus*. Anim. Genet..

[22] Morris C.A., Cullen N.G., Hickey S.M., Dobbie P.M., Veenvliet B.A., Manley T.R., Pitchford W.S., Kruk Z.A., Bottema C.D.K., Wilson T. (2006). Genotypic effects of calpain 1 and calpastatin on the tenderness of cooked M. longissimus dorsi steaks from Jersey×Limousin, Angus and Hereford-cross cattle. Anim. Genet..

[23] Nattrass G.S., Cafe L.M., McIntyre B.L., Gardner G.E., McGilchrist P., Robinson D.L., Wang Y.H., Pethick D.W., Greenwood P.L. (2014). A post-transcriptional mechanism regulates calpastatin expression in bovine skeletal muscle. J. Anim. Sci..

[24] Wittwer C.T., Reed G.H., Gundry C.N., Vandersteen J.G., Pryor R.J. (2003). High-resolution genotyping by amplicon melting analysis using LCGreen. Clin. Chem..

[25] Reed G.H., Kent J.O., Wittwer C.T. (2007). High-resolution DNA melting analysis for simple and efficient molecular diagnostics. Pharmacogenomics.

[26] R Core Team (2025). R: A Language and Environment for Statistical Computing.

[27] Liew M., Pryor R., Palais R., Meadows C., Erali M., Lyon E., Wittwer C. (2004). Genotyping of single-nucleotide polymorphisms by high-resolution melting of small amplicons. Clin. Chem..

[28] Van Eenennaam A.L., Li J., Thallman R.M., Quaas R.L., Dikeman M.E., Gill C.A., Franke D.E., Thomas M.G. (2007). Validation of commercial DNA tests for quantitative beef quality traits. J. Anim. Sci..

[29] White S.N., Casas E., Wheeler T.L., Shackelford S.D., Koohmaraie M., Riley D.G., Chase C.C., Johnson D.D., Keele J.W., Smith T.P.L. (2005). A new single nucleotide polymorphism in CAPN1 extends the current tenderness marker test to include cattle of *Bos indicus*, *Bos taurus*, and crossbred descent. J. Anim. Sci..

[30] Carvalho M., Eler J.P., Bonin M.N., Rezende F.M., Biase F.H., Meirelles F.V., Regitano L.C.A., Coutinho L.L., Balieiro J.C.C., Ferraz J.B.S. (2017). Genotypic and allelic frequencies of gene polymorphisms associated with meat tenderness in Nellore beef cattle. Genet. Mol. Res..

